# Exogenous pentraxin-3 inhibits the reactive oxygen species-mitochondrial and apoptosis pathway in acute kidney injury

**DOI:** 10.1371/journal.pone.0195758

**Published:** 2018-04-19

**Authors:** Hyung Ho Lee, Sook Young Kim, Joon Chae Na, Young Eun Yoon, Woong Kyu Han

**Affiliations:** 1 Department of Urology, National Health Insurance Service Ilsan Hospital, Gyeonggi-do, Korea; 2 Department of Urology, Urological Science Institute, Yonsei University College of Medicine, Seoul, Korea; 3 Department of Urology, Hanyang University College of Medicine, Seoul, Korea; 4 Brain Korea 21 PLUS Project for Medical Science, Yonsei University College of Medicine, Seoul, Korea; National Institutes of Health, UNITED STATES

## Abstract

Pentraxin-3 (PTX3) is a long-form member of the pentraxin family of proteins that has been studied in inflammatory diseases and in various organs. We found that PTX3 protects kidney cells during ischemia and proinflammatory acute kidney injury. The aim of this study was to develop an *in vitro* experimental model of acute kidney injury and to analyze the protective mechanism of exogenous recombinant PTX3. In this study, cells of the HK-2 renal tubular cell line were treated with a calcium ionophore (A23187), which induced injury by increasing intracellular calcium concentrations and inducing calpain activity and the generation of reactive oxygen species. Exposure of cells to PTX3 significantly attenuated these effects. In addition, the activity of caspase-3 and PARP-1 were decreased in ischemic cells exposed to exogenous recombinant PTX3. PTX3 stabilized the mitochondrial membrane potential and suppressed apoptosis, resulting in the protection of renal tubular cells from ischemic injury.

## Introduction

The pentraxins (PTXs) are a superfamily of multifunctional multimeric proteins divided into short (C-reactive protein and serum amyloid P component) and long (PTX3) forms.[1] PTX3 is rapidly induced by several stimuli in different cell types.[2] Peripheral blood leukocytes and myeloid dendritic cells release PTX3 in response to proinflammatory cytokines (interleukin [IL]-1 and tumor necrosis factor-α [TNF-α]), to agonists of Toll-like receptors (TLRs), and to stimulation with microbial components.[2] Different signaling pathways can affect PTX3 production depending on the cell and/or stimulus types. PTX3 binds the C1q complex and activates the classical complement pathway.[2] PTX3 also participates in the clearance of apoptotic cells and several microorganisms.[1] Furthermore, PTX3 has been known to exert counteracting effects against interferon-gamma (IFN-γ) and IL-10 in inflammatory reactions. PTX3 has been identified as a key factor in the host defense against certain fungal, bacterial, and viral infections.

PTX3 levels are very low in the serum and tissues of normal subjects but rapidly increase in response to inflammatory stimulation in a wide range of diseases, including infectious, autoimmune, and degenerative disorders.[3,4] Results from clinical tests suggest that elevated PTX3 levels may serve as a sensitive marker for determining early diagnoses and prognoses of certain severe illnesses, such as acute myocardial infarction.[5,6] The role of PTX3 in inflammatory conditions was investigated *in vivo* using transgenic mice carrying multiple copies of *Ptx3*.[7] Lipopolysaccharide exposure induces the overexpression of PTX3 in these mice, which significantly improves their survival. In contrast, PTX3-deficient animals exhibit greater myocardial damage associated with an increased neutrophil infiltration in a model of cardiac ischemia/reperfusion injury.[8]

Levels of PTX3 levels are also increased in acute respiratory distress syndrome, in cardiovascular disease, in a variety of atherosclerotic diseases, and in kidney disease. Interestingly, it was reported that PTX3 affects the counterbalancing mechanism for mononuclear phagocytes in TNF-induced acute kidney injury (AKI).[9] The authors found that PTX3 inhibited leukocyte adhesion and transmigration and relieved sterile renal inflammation. Eventually, PTX3 contributed to the recovery of injured tubular cells.

Clinical cases of AKI frequently result from renal ischemia-reperfusion injury (IRI), with an incidence exceeding 50% after major cardiac, hepatobiliary, or aortic surgery.[10,11] Renal IRI, which results from cardiopulmonary bypass, partial nephrectomy, and renal transplantation, is an emerging clinical problem, as acute renal injury can result in end-stage renal disease.[12] Ischemic AKI is frequently complicated by multiorgan dysfunction, systemic inflammation, sepsis, and death.[10] Renal cells are damaged during AKI and may undergo apoptosis, or programmed cell death, which is an evolutionarily conserved and highly regulated process involving a series of molecular events.[13,14]

Biochemical and metabolic alterations that occur during IRI include the generation of reactive oxygen species (ROS), decreasing ATP levels, increasing inflammatory mediators, and rapid restoration of a physiological pH, which in turn increases intracellular sodium concentrations and leads to an overload of intracellular and mitochondrial calcium. Reperfusion injury is mediated by the interaction of these factors, which opens the mitochondrial permeability transition pore and initiates cell death pathways.[15] ROS induce oxidative stress and affect various cell signaling pathways, including apoptotic pathways, after long-term and irreversible accumulation.[16] Indeed, ROS can increase intracellular calcium levels and influence mitochondrial function, leading to the activation of a pro-apoptotic protein.[14,16] Ischemia, inflammatory damage, and ROS-induced injury lead to cell death via activation of either the caspase-3 pathway or a recently identified caspase-independent pathway mediated by the activation of poly(ADP-ribose) polymerase-1 (PARP-1).[13,17]

With this in mind, we speculated that PTX3 might regulate postischemic tissue inflammation and injury. We utilized a model of ischemic AKI induced by Ca^2+^ overload and hypoxia to determine whether administration of exogenous recombinant PTX3 protects against ischemic AKI *in vitro* and to examine the mechanism for recovery.

## Material and methods

### Cell culture

Institutional Review Board at Yonsei University (College of Medicine, Yonsei University), and the study was approved by the ethics committee. HK-2 cell, which are human renal proximal tubular epithelial cell, were obtained from the American Type Culture Collection (ATCC, Manassas, VA, USA).HK-2 cells were cultured in keratinocyte serum-free medium (Gibco, Grand Island, NY, USA) supplemented with 0.05 mg/mL bovine pituitary extract, 5 ng/mL human recombinant epidermal growth factor, 10% fetal bovine serum, and 1% penicillin/streptomycin in an atmosphere of 5% CO_2_/95% O_2_ at 37°C. Cells were subcultured every 7 d using 0.02% ethylenediaminetetraacetic acid and 0.05% trypsin. The medium was replaced with fresh medium every 2 d.

### Cell viability

HK-2 cells (2×10^4^ cells/mL) in 96-well culture plates were treated with various doses of recombinant human PTX3 (Sino Biological, Inc., China) for 24 h. Cell viability was assessed using the Cell counting kit-8 (Dojindo Laboratories, Kumamoto, Japan) in accordance with the manufacturer’s instructions.Briefly,2-(2-methoxy-4-nitrophenyl)-3-(4-nitrophenyl)-5-(2,4-disulfophenyl)-2H-tetrazolium monosodium salt was added to each well, and wells were incubated for 1 h. The formation of a water-soluble formazan product in the medium was determined using a Beckman Coulter Microplate reader at 450 nm. Treatment with TGF-β was used as a positive control. For hypoxic treatment, HK-2 cells were incubated overnight in a hypoxic chamber (Tapei/Espec, Osaka, Japan); for ischemic treatment, cells were incubated with the Ca^2+^ ionophore, A23187 (Sigma).

### Intracellular calcium measurement

HK-2 cells in 12-well culture plates were loaded with a fluo-4/NW Ca^2+^ indicator (Molecular Probes, Belgium) and incubated at 37°C for 45 min according to the manufacturer’s instructions. The fluorescent images were acquired using a fluorescent microscope (Olympus America, Melville, NY).

### Calpain activity assay

To detect the activity of calpain in cells, we used a calpain activity fluorometric assay kit (Biovision, CA, US) according to the manufacturer’s instructions. Briefly, a calpain substrate, Ac-LLY-AFC, was added to the supernatants of cell lysate extracts, which were incubated at 37°C for 1 h in the dark. The cleaved substrates were excited at 400 nm and analyzed at 505 nm using ta fluorometer (Varioskan Flash 3001; Thermo Fisher Scientific, Vantaa, Finland).

### ROS assay

HK-2 cells in 12-well culture plates were stained with DCF (Molecular Probes, Belgium) and incubated at 37°C for 45 min according to the manufacturer’s instructions. The fluorescent images were acquired using a fluorescent microscope (Olympus America, Melville, NY).

### MMP assay

HK-2 cells in a glass-bottom dish (glass diameter, 10 mm) were treated with the mitochondrial probe 5,5′,6,6′-tetrachloro-1,1′,3,3′-tetraethyl benzimidazolyl-carbocyanine iodide (JC-1; Cayman Chemical, Ann Arbor, MI) and incubated at 37°C for 20 min. Healthy cells with mainly JC-1 aggregates were detected with excitation at 540 nm and emission at 570 nm, and apoptotic or unhealthy cells with mainly JC-1 monomers were detected with excitation at 485 nm and emission at 535 nm using a confocal microscope (LSM Meta 700; Carl Zeiss, Oberkochen, Germany).

### TUNEL staining

Apoptotic cells were detected by TUNEL staining using a TACS 2 TdT-FITC *in situ* apoptosis detection kit (Trevigen, Inc., Gaithersburg, MD) according to the manufacturer’s instructions. Images were taken on a confocal microscope (LSM Meta 700; Carl Zeiss, Oberkochen, Germany) and analyzed with LSM Image Browser software.

### Western blotting

Total cellular protein extracts were prepared on ice using a PRO-PREP protein extract solution (Intron, Seoul, Korea). Cell lysates were loaded onto a sodium dodecyl sulfate-polyacrylamide gel and transferred to a polyvinylidene fluoride membrane for 1 h. Membranes were incubated 4°C overnight with cleaved PARP, active caspase-3, PTX3, or GAPDH primary antibodies (Abcam) diluted 1:1000 with 5% bovine serum albumin in Tris-buffered saline-Tween 20 (TBS-T). After incubation, the membranes were washed with TBS-T, and secondary antibodies (1:10000; horseradish peroxidase-conjugated anti-mouse or anti-rabbit IgG) were applied at room temperature for 1 h. Labeled bands were detected using a West Pico chemiluminescence kit (Thermo Scientific, Rockford, IL, USA).

### Statistical analysis

Quantitative values are expressed as means ± S.E.M. Statistical differences were determined using the Kruskal-Wallis test and Mann-Whitney U-test. A *p*-value of <0.05 was considered statistically significant. Statistical analyses were performed using SPSS software version 23.0 (IBM SPSS Statistics, IBM Corp., Armonk, NY, USA).

## Results

### Exogenous PTX3 increases cell viability at low concentration

**Fig 1:**
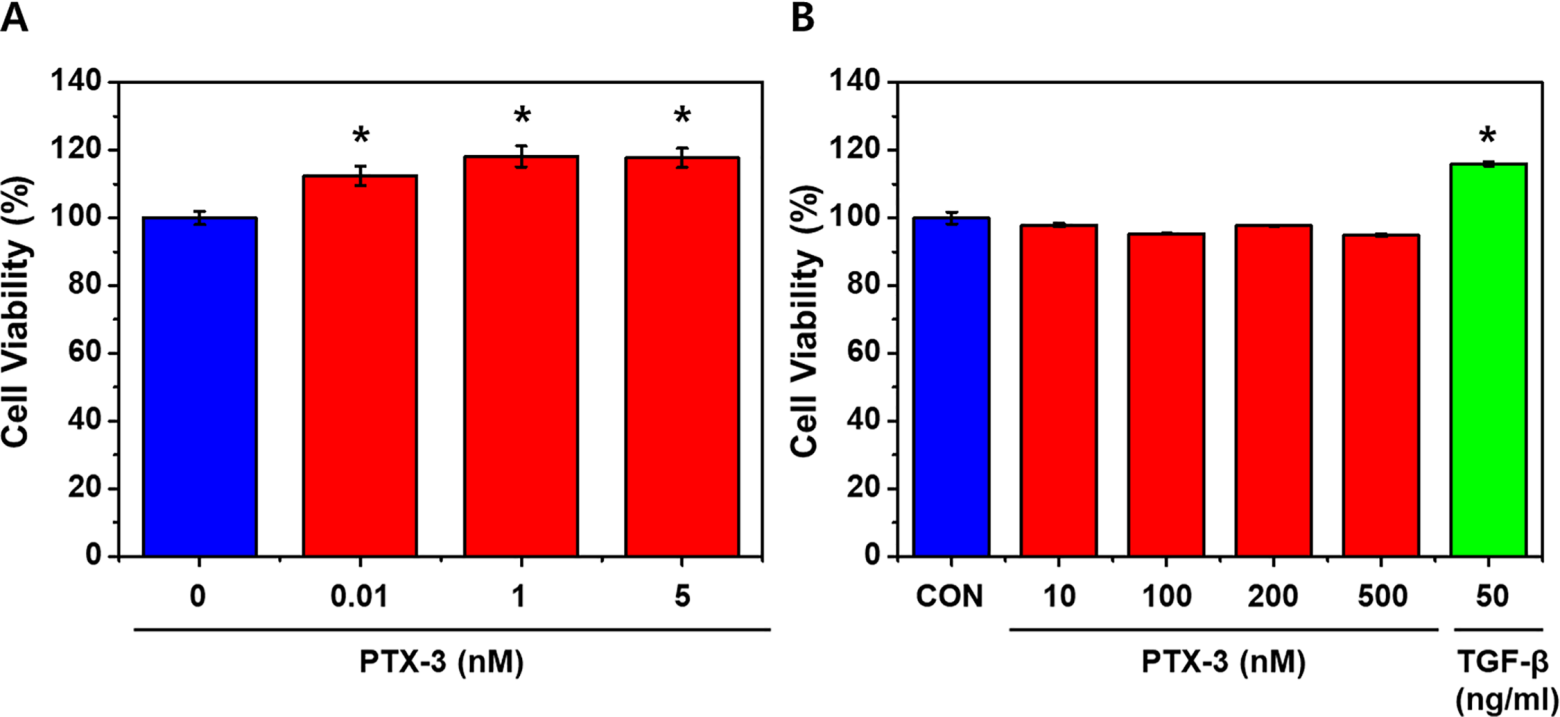
The viability of HK-2 cells with exogenous PTX3. Treatments with low (A) and high (B) doses of exogenous PTX3 (P) were compared. Cont, control; T50, 50 ng/mL TGF-β (positive control). Data are represented as means ± s.e.m from each HK-2 group (n = 3). *p < 0.05. 'CON' is abbreviated 'control' which is treated nothing.

The viability of HK-2 cells increased significantly with the addition of 1, 5 nM of PTX3 (Fig 1A)(*p<0.05). However, increasing doses did not further effect on cell viabilities (Figs 1A and 1B). The viabilities of cells treated with high doses (up to 500 nM) of PTX3 were also compared with those of untreated cells and cells treated with 50 ng/ml TGF-β, as negative and positive controls, respectively. The highest cell viability was observed in cells treated with 1 nM PTX3 (Fig 1B).

### PTX3 protects cells from ischemic/hypoxic injury

**Fig 2:**
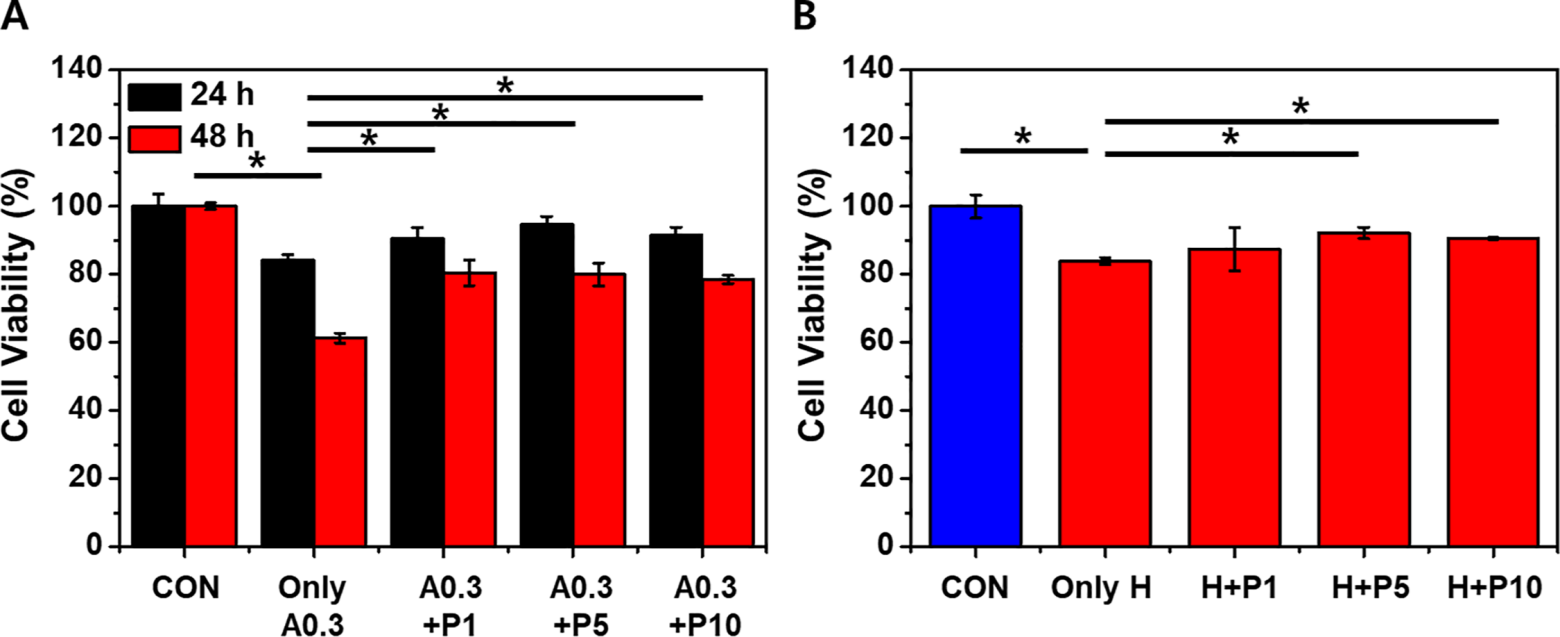
Exogenous PTX3 improves the viability of HK-2 cells after ischemic and hypoxic injuries. Cell viabilities were measured with and without PTX3 at 1, 5, or 10 nm (P1, P5, and P10, respectively) (A) 24 and 48 h after treatment with 0.3 μg/mL A23187 (A0.3) and (B) after exposure to a hypoxic environment (H). We show that PTX3 provides significant cell viability increasing against HK-2 cell injury after 48hr. Values are expressed as means ± s.e.m of at least four independent experiments, *p < 0.05 (n = 4). 'CON' is abbreviated 'control' which is treated nothing.

The ischemic injury was modeled in HK-2 cells by treatment with the calcium ionophore A23187 (0.3 μg/mL) or by exposure to a hypoxic chamber. The viabilities of cells treated with A23187 were significantly decreased compared to those of the control groups at 24 and 48 h (Fig 2A)(*p< 0.05). However, the viability after 48 h of cells that also received PTX3 was significantly higher than the group that received only A23187 (Fig 2A)(*p< 0.05). The viabilities of cells coadministered 1, 5, and 10 nM PTX3 did not differ. Cell viabilities were significantly reduced by exposure to the hypoxic chamber (Fig 2B). However, treatment with 5 nM and 10 nM exogenous PTX3 after hypoxic injury increased cell viabilities compared to hypoxic injury only (Fig 2B). Cell viabilities after hypoxia did not differ between cells treated with 1, 5, and 10 nM PTX3.

### PTX3 reduces [Ca^2+^]_i_ in ischemic cells

**Fig 3:**
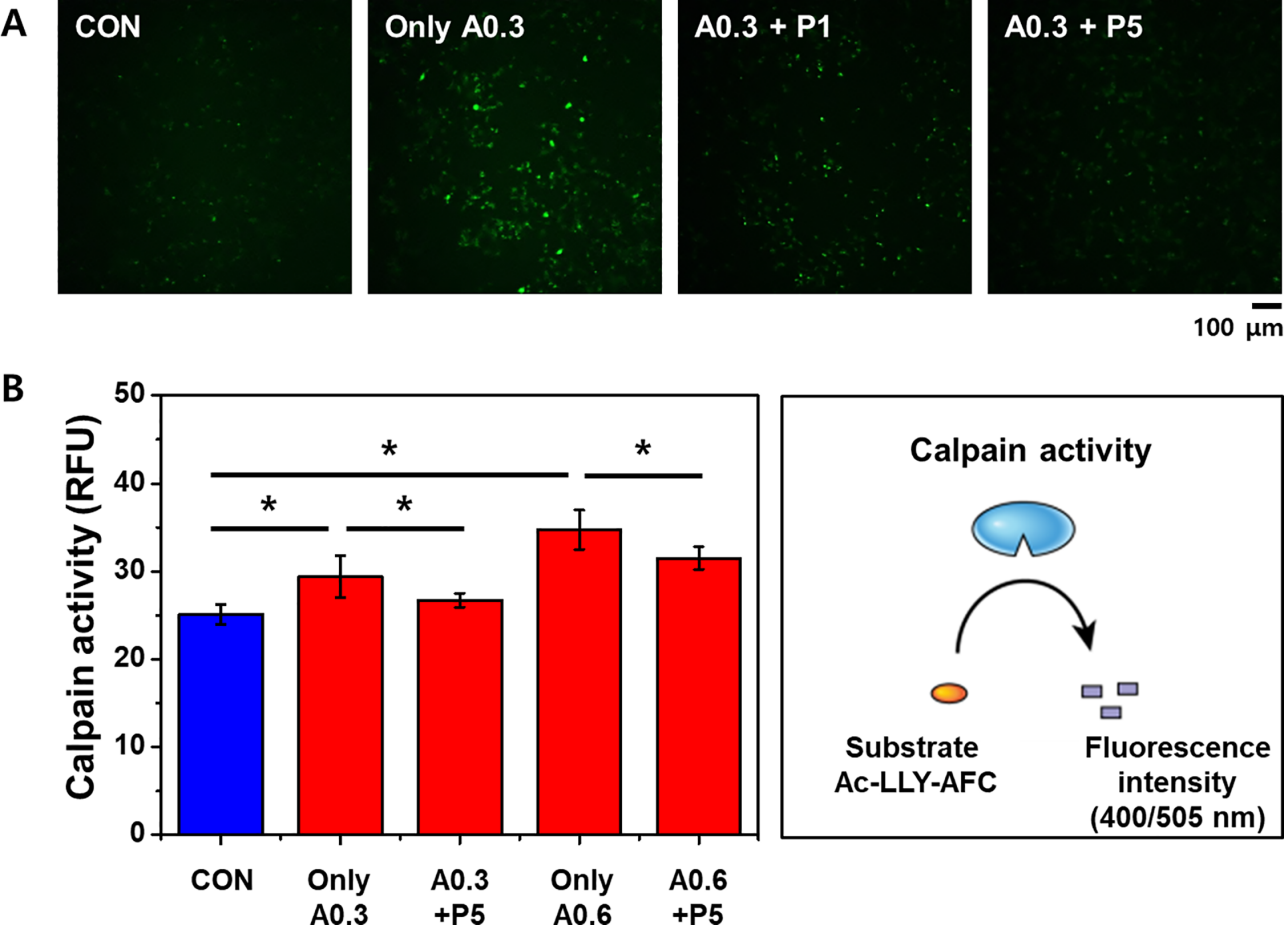
Exogenous PTX3 attenuates calcium responses to ischemic injury. (A) Intracellular calcium indicated by fluo-4 fluorescence was enhanced in HK-2 cells treated with 0.3 μg/mL A23187 and reduced by the addition of 1 and 5 nM PTX3 (P1 and P5, respectively). (B) Calpain activity as measured by fluorimetry increased in cells treated with 0.3 μg/mL and 0.6 μg/mL A23187 (A0.3 and A0.6, respectively), which was attenuated by treatment with PTX3. Results represent the mean ± s.e.m of four independent experiments (n = 4). The statistical signification was marked as *p < 0.05 respectively, compared with control. 'CON' is abbreviated 'control' which is treated nothing.

In HK-2 cells treated with A23187, intracellular Ca^2+^ levels ([Ca^2+^]_i_) and calpain activity were determined after exposure to exogenous PTX3. By using microscopy to measure fluo-4 fluorescence, we found that treatment with A23187 increases [Ca^2+^]_i_ in HK-2 cells (Fig 3A). However, [Ca^2+^]_i_ decreased in cells also treated with exogenous PTX3 (5 nM). We also assessed the activity of calpain, an indicator of apoptotic cell death, by fluorimetry. As measured by relative fluorescence units (RFU), calpain activity in HK-2 cells increased with increasing A23187 concentrations (Fig 3B). Furthermore, these increases of clapain activity were attenuated by treatments with 5 nM PTX3 (Fig 3B).

### PTX3 reduces the production of ROS in ischemic cells

**Fig 4:**
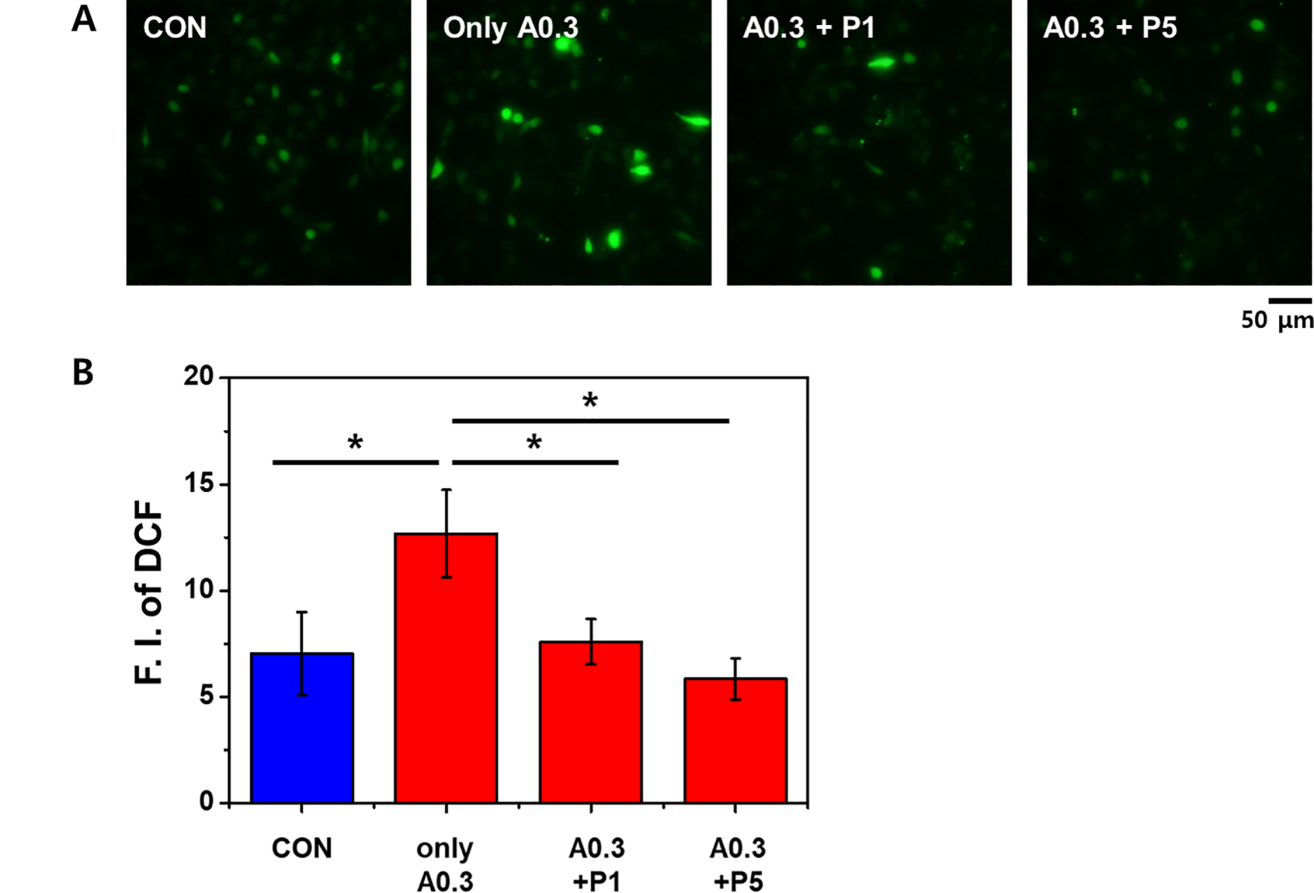
(A) DCF staining for ROS increased with A23187 treatment and was attenuated with exposure to 1 and 5 nM PTX3 (P1 and P5, respectively). (B) ROS activity as measured by fluorimetry increased(F.I. of DCF) in cells treated with 0 nM/mL and 5 nM/mL PTX3 (A0.3, A0.3+P1 and A0.3+P5, respectively), which was attenuated by treatment with PTX3. *p < 0.05 versus A23187 (0.3μg/mL) subjected to administrate PTX3. The statistical signification was marked as * for p < 0.05 compared with the control. 'CON' is abbreviated 'control' which is treated nothing.

To evaluate the generation of intracellular ROS, HK-2 cells treated with A23187 and exogenous PTX3 were stained with 2',7'-dichlorodihydrofluorescein diacetate (DCF). The fluorescence intensity was significantly lower in A23187-treated cells that were exposed to 1 and 5 nM PTX3 compared with the intensity in cells treated with A23187 alone (Fig 4A). As measured by relative fluorescence units (RFU), DCF staining in HK-2 cells increased with A23187 treated group (Fig 4B)(*p< 0.05). Moreover, the intensity of DCF staining was attenuated by the higher PTX3 dose, significantly (Fig 4B)(*p< 0.05).

### PTX3 retains MMP in ischemic cells

**Fig 5:**
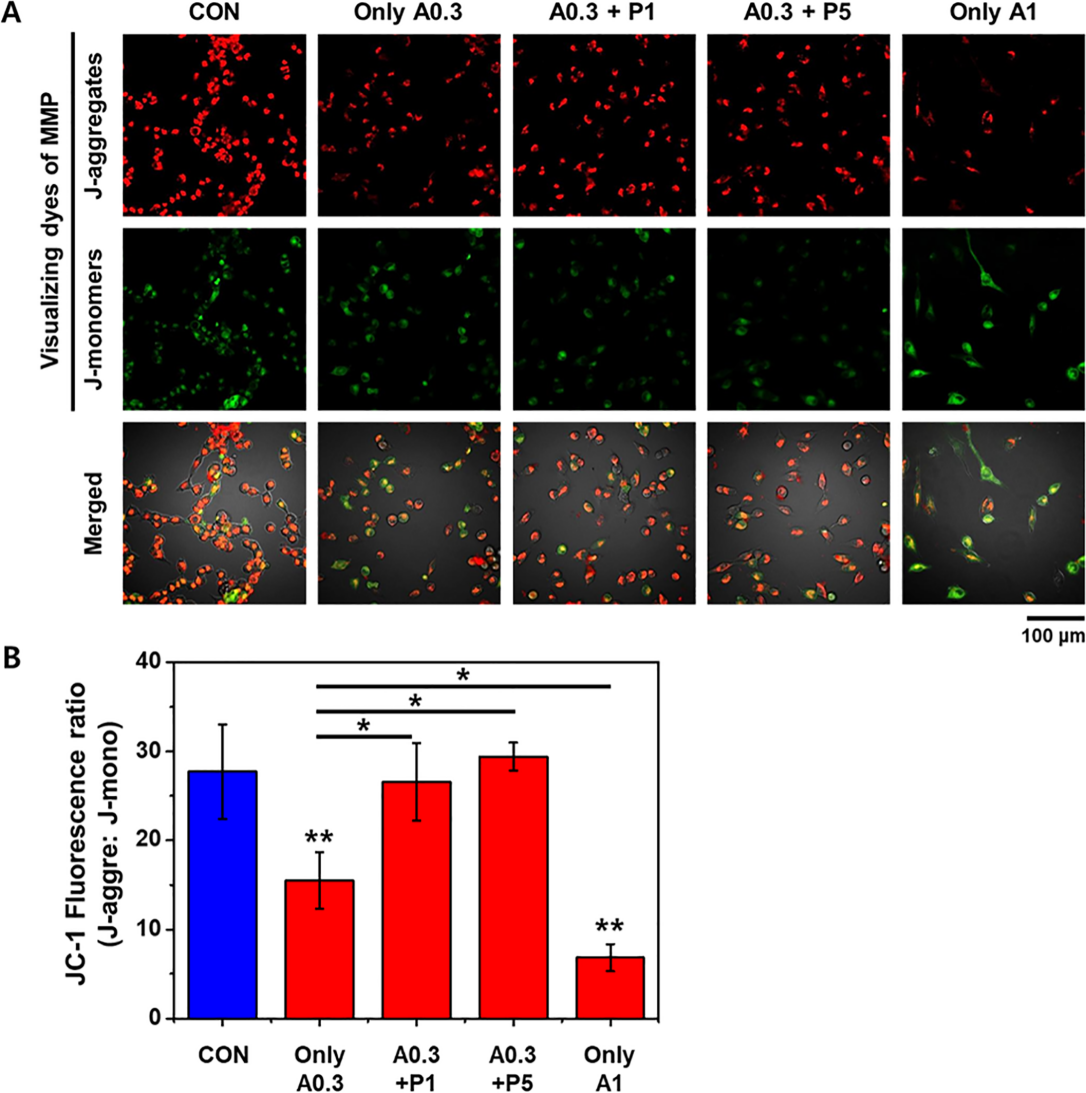
(A) Mitochondrial membrane potentials were visualized by the intensity of fluorescing JC-1 aggregates (red) and monomers (green), which indicate high and low potentials, respectively, after treatment with 0.3 or 1 μg/mL A23187 (A0.3 and A1, respectively) and exposure to PTX3. (B) Numerical data were expressed as % Red/Green fluorescence cells which were increased with increasing doses of PTX3. Data is representative of five independent experiments and expressed as means ± s.e.m, *p < 0.05 as compared with their respective A23187 (0.3μg/mL) only. **p < 0.001 as compared control group and A23187administrated group (0.3μg/mL and 1μg/mL). 'CON' is abbreviated 'control' which is treated nothing.

The effect of ROS generation on the mitochondrial membrane potential (MMP) in HK-2 cells was measured via JC-1 fluorescence using confocal microscopy. Membrane potential (ΔΨm) is used for characterization of cellular metabolism, viability, and apoptosis. The loss of mitochondrial membrane potential (ΔΨm) is a hallmark of apoptosis. Fig 5 showing JC-1 red, JC-1 green and merge image. The JC-1 green fluorescence indicates a decrease in mitochondrial membrane potential, an early event in apoptosis. Increased concentrations of PTX3 attenuated the loss of mitochondrial membrane potential. A high MMP is depicted as red fluorescence from aggregates of JC-1, whereas a low MMP is depicted as green fluorescence from JC-1 monomers. As shown in Fig 5, control HK-2 cells exhibited strong red fluorescence, indicating the cells had a high MMP. However, the red fluorescence decreased after exposure to A23187 (0.3 μg/mL), with the corresponding green fluorescence indicating a low MMP. Moreover, the intensity of the green fluorescence increased while the red fluorescence decreased when the concentration of A23187 was increased to 1 μg/mL. The addition of exogenous PTX3 enabled A23187-treated cells to retain their MMP, exhibited as greater red fluorescence with reduced green fluorescence, corresponding to a high MMP (Fig 5A).

The JC-1 fluorescence ratio was significantly lower in A23187-treated cells that were exposed to 0.3 and 0.6 μg/mL, compared with the intensity in cells untreated (Fig 5B) (** p<0.01).

As shown from quantitative data, the Red/Green-fluorescence cells ratio were found to be 15.48%, 26.57%, 29.42% and 6.82% at A23187 0.3μg/mL only, 0.3μg/mL (A23187) + 1nM (PTX3), 0.3μg/mL (A23187) + 5nM (PTX3) and A23187, 1 μg/mL only treatment, respectively (Fig 5B)(*p<0.05).

### PTX3 reduces apoptosis in ischemic cells

**Fig 6:**
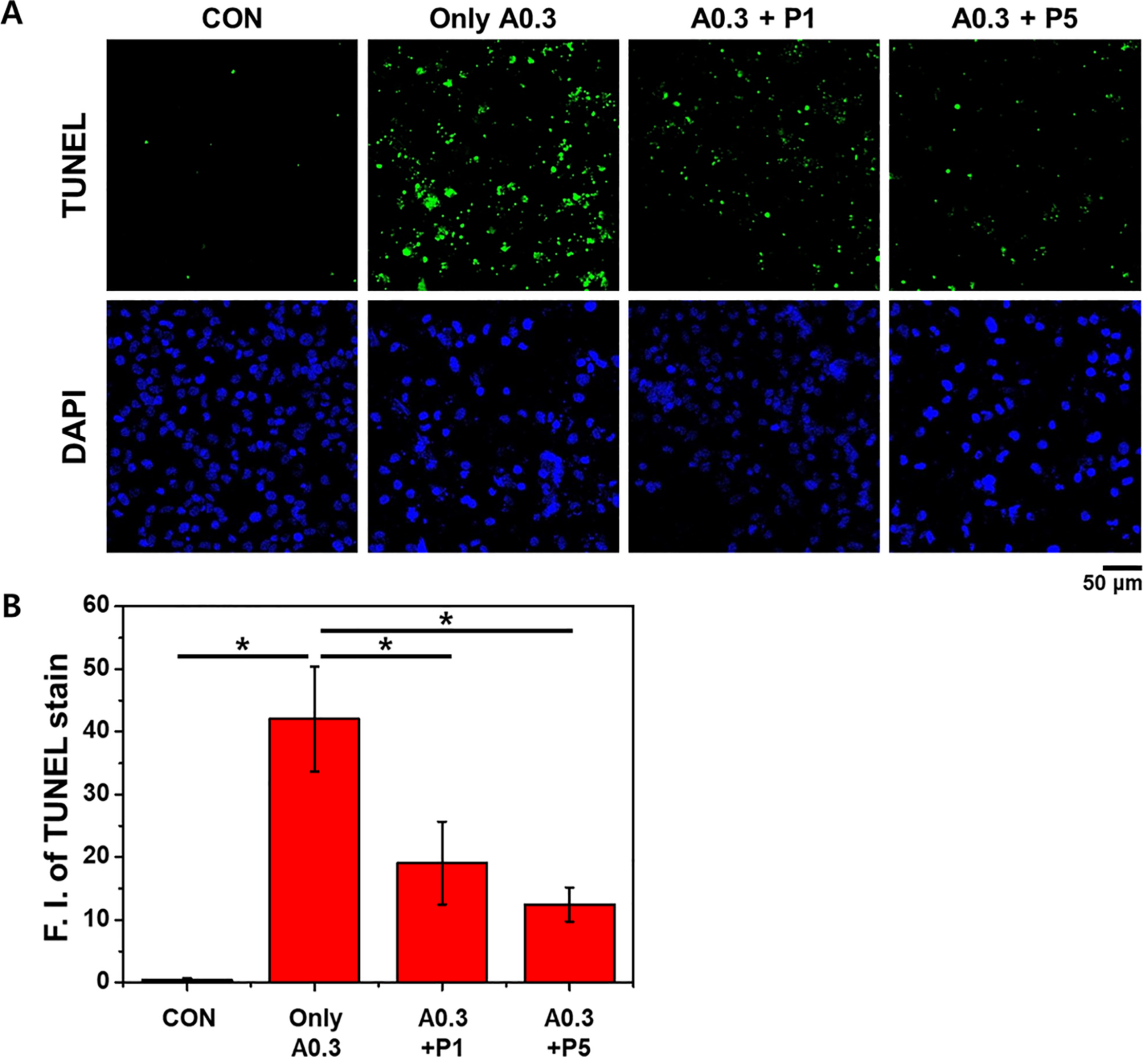
Treatment of HK-2 cells with 0.3 μg/mL A23187 (A0.3) induces apoptosis as determined by TUNEL assays. Representative micrographs (A) and quantification (B) of TUNEL staining revealed that apoptotic processes were attenuated in ischemic cells exposed to 1 and 5 nM PTX3 (P1 and P5, respectively). Numerical data were expressed as mean intensity apoptotic cells respective to their control. Data expressed means ± s.e.m, *p < 0.05 as compared with their respective A23187 (0.3μg/mL) only. 'CON' is abbreviated 'control' which is treated nothing.

We performed terminal deoxynucleotidyl transferase-mediated digoxigenin-deoxyuridine nick-end labeling (TUNEL) to identify apoptotic cells. Apoptosis of HK-2 cells was induced by A23187 (Fig 6A). However, treatment of these ischemic cells with 1 or 5 nM PTX3 attenuated this response. These results were verified by quantification of the fluorescence intensities, which showed that cells treated with A23187 had the highest average fluorescence intensity and that this was reduced by exposures to increasing concentrations of PTX3 (Fig 6B)(*p<0.05).

### PTX3 reduces the activity of caspase-3 and PARP-1 in ischemic cells

**Fig 7:**
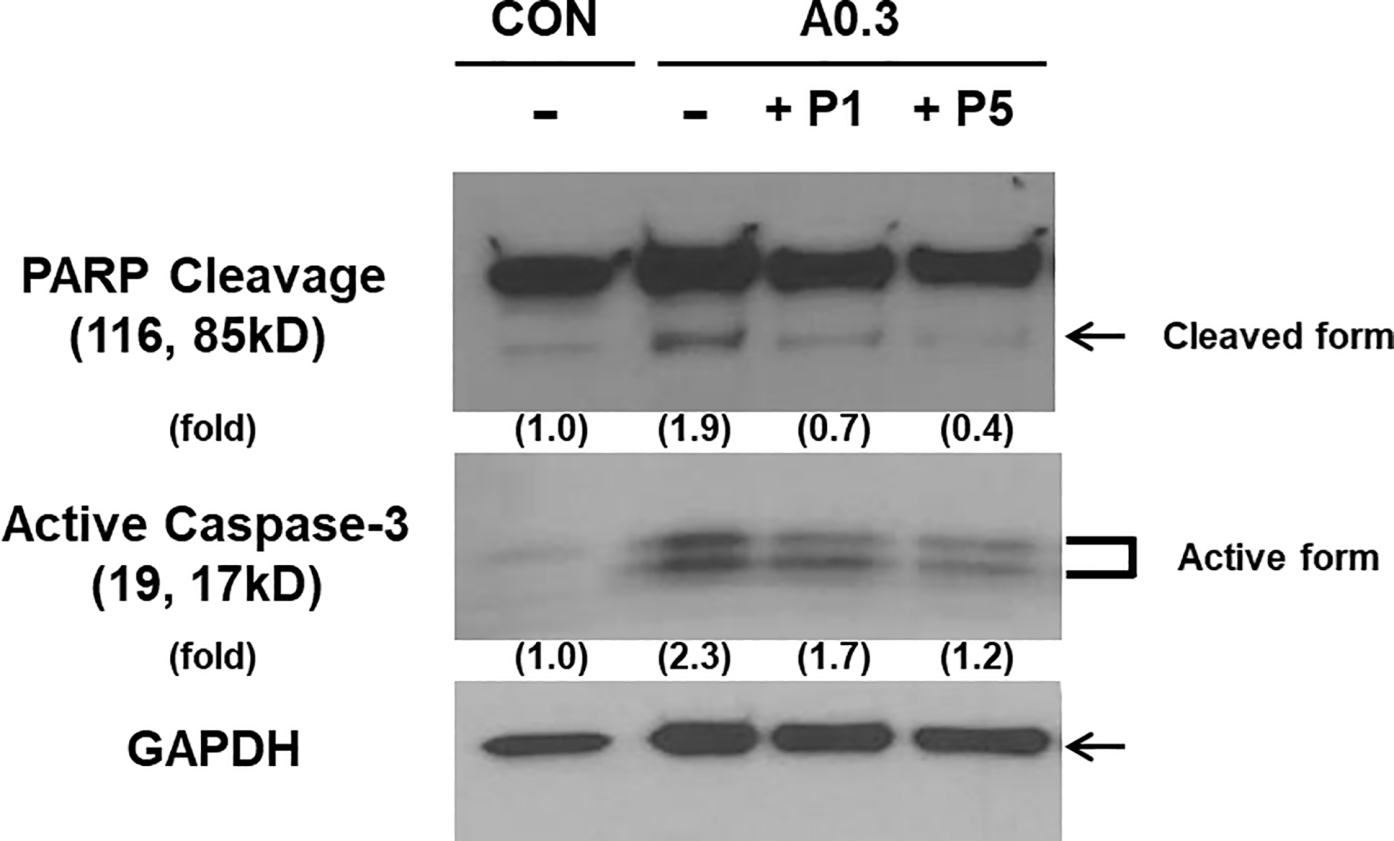
Active caspase-3 and PARP cleavage were detected by Western blotting of supernatants from untreated cells and cells treated with 0.3 μg/mL A23187. The corresponding increases in PARP cleavage, and active caspase-3 were attenuated in cells treated with 1 or 5 nM PTX3 (P1 and P5, respectively). GAPDH was used as a loading control. 'CON' is abbreviated 'control' which is treated nothing.

ROS-induced injury can lead to cell death via caspase-3 and caspase-independent (mediated by PARP-1 activation) pathways.[17] To clarify which pathway plays a role in our model of ischemia, we performed Western blotting for caspase-3 and the DNA repair protein PARP-1. The treatment of HK-2 cells with 0.3 μg/mL A23187 increased the level of active caspase-3, which is consistent with the initiation of apoptosis (Fig 7). However, this increase in active caspase-3 was attenuated in cells exposed to PTX3. We also observed that A23187 treatment resulted in cleavage of PARP-1, which was similarly attenuated in cells exposed to PTX3. Thus, A23187 stimulates caspase-3 and caspase-independent pathways, both of which are suppressed after exposure to PTX3.

## Discussion

Acute IRI is one of the most common acute kidney injuries that occurs during partial nephrectomy or other ischemic lesions. However, the influence of secondary inflammatory responses on kidney cells has not yet been clarified. In AKI, TLR-4 was shown to act as a major maladaptive high-mobility group protein B1 receptor and to be involved in the positive regulation of PTX3.[18] Accordingly, it appears that PTX3 may have a proinflammatory role. In this respect, PTX3 may be considered as an adverse factor to IRI.[18] However; it was also hypothesized that PTX3 plays an anti-inflammatory role in IRI of kidney cells on the basis of its cardioprotective effect in acute myocardial infarction.[19] Furthermore, PTX3 reportedly counteracts TNF-induced proinflammatory cytokines in kidney cells.[1,9] Previously reported data document that the acute-phase protein PTX3 is induced systemically and inside postischemic kidneys, which limits leukocyte adhesion and transmigration.[20] This mechanism prevents overactivation of the immune system and helps resolve sterile inflammation as a prerequisite for rapid and effective tubular repair. Thus, two contradictory findings in the acute injury of renal cells indicate that PTX3 may play a role in kidney cell damage. Previous reports also showed that innate immune effector cells largely contribute to postischemic renal inflammation and AKI. Oxidative stress induces the expression of PTX3 inside the kidney, which suppresses leukocyte recruitment to the ischemic injury.[9,21] This induction represents an endogenous mechanism to limit unnecessary IR injury. However, a later role for PTX3 in suppressing renal inflammation remains to be defined. We conducted this study to determine how PTX3 influences renoprotection and cell signaling after IRI.

Stimulation with TNF-α and IL-1β is reported to strongly induce the production of PTX3.[22] We first confirmed that proinflammatory signals (TNF-α and IL-1β) induce the expression of PTX3 in a kidney cell line. Thus, PTX3 secreted from injured tubular cells could potentially affect a specific region during injury.

The effect of exogenous PTX3 on cell viability was confirmed (Fig 1). This was an experiment to find the concentration of PTX3 with cell protective effect. Exogenous PTX3 increased cell viability at low concentrations. However, there was no increase in cell viability at high concentrations (Fig 1). The purpose of Fig 1 was a preliminary experiment to determine the extent to which PTX3 concentration should be administered in subsequent experiments to demonstrate its efficacy without cytotoxicity. Fig 1 shows that cell viability was affected by lower dose PTX3. In our further experiments, we tried to reveal the pathway of the protective effects of PTX3 with a volume of 1–5 nM

To establish an *in vitro* model of ischemic injury, HK-2 cells were treated with the calcium ionophore A23187 (0.3 μg/mL), and these cells were then exposed to various concentrations of PTX3. After 48 hours, cells exposed to PTX3 exhibited higher cell viability than those exposed to ischemic (or hypoxic) injury alone (Fig 2).

Our results confirm the protective effect of PTX3 and indicate that PTX3 suppresses Ca^2+^ influx and subsequent calpain activity. High intracellular concentrations of calcium induce cytochrome c release and apoptosome formation and activate calpain, and caspase-3.[23] Calpains can affect mitochondrial function and are essential for apoptosis in human microvascular endothelial cells.[24] Much of the mitochondrial calpain research performed to date has focused on its proapoptotic role in the cleavage of caspase-3.[25] Our results show that ischemia caused by A23187 increased [Ca^2+^]_i_, as expected, and that exposure to PTX3 attenuated this (Fig 3). Consequently, calpain activity was also decreased, as was the production of ROS, in cells exposed to PTX3 (Fig 4).

The ROS-induced change in mitochondrial function was observed in ischemic HK-2 cells as a decrease in the fluorescence from JC-1 aggregates, indicative of an increased MMP. However, the exposure of these cells to PTX3 stabilized the MMP. Moreover, MMP was stabilized as PTX3 increased. The changes in MMP were mirrored by the increase in apoptosis in ischemic cells, with protection proffered by exposure to PTX3 (Figs 5 and 6).

One of the signaling cascades most commonly involved in apoptosis is the activation of a highly specialized family of caspases. Activated caspases initiate cell death by cleaving and activating effector caspases[26] and PARP-1, the cleavage of which is considered a hallmark of apoptosis.[27,28] Ischemic injury in HK-2 cells induced by A23187 resulted in increases in the active form of caspase-3 and cleavage of PARP-1. Interestingly, these apoptotic indications were attenuated in cells exposed to PTX3. These results demonstrate that A23187 induced apoptosis in HK-2 cells through activation of caspase-3 and PARP and that PTX protects cells against calcium-mediated mitochondrial apoptosis pathways (Fig 7).

**Fig 8:**
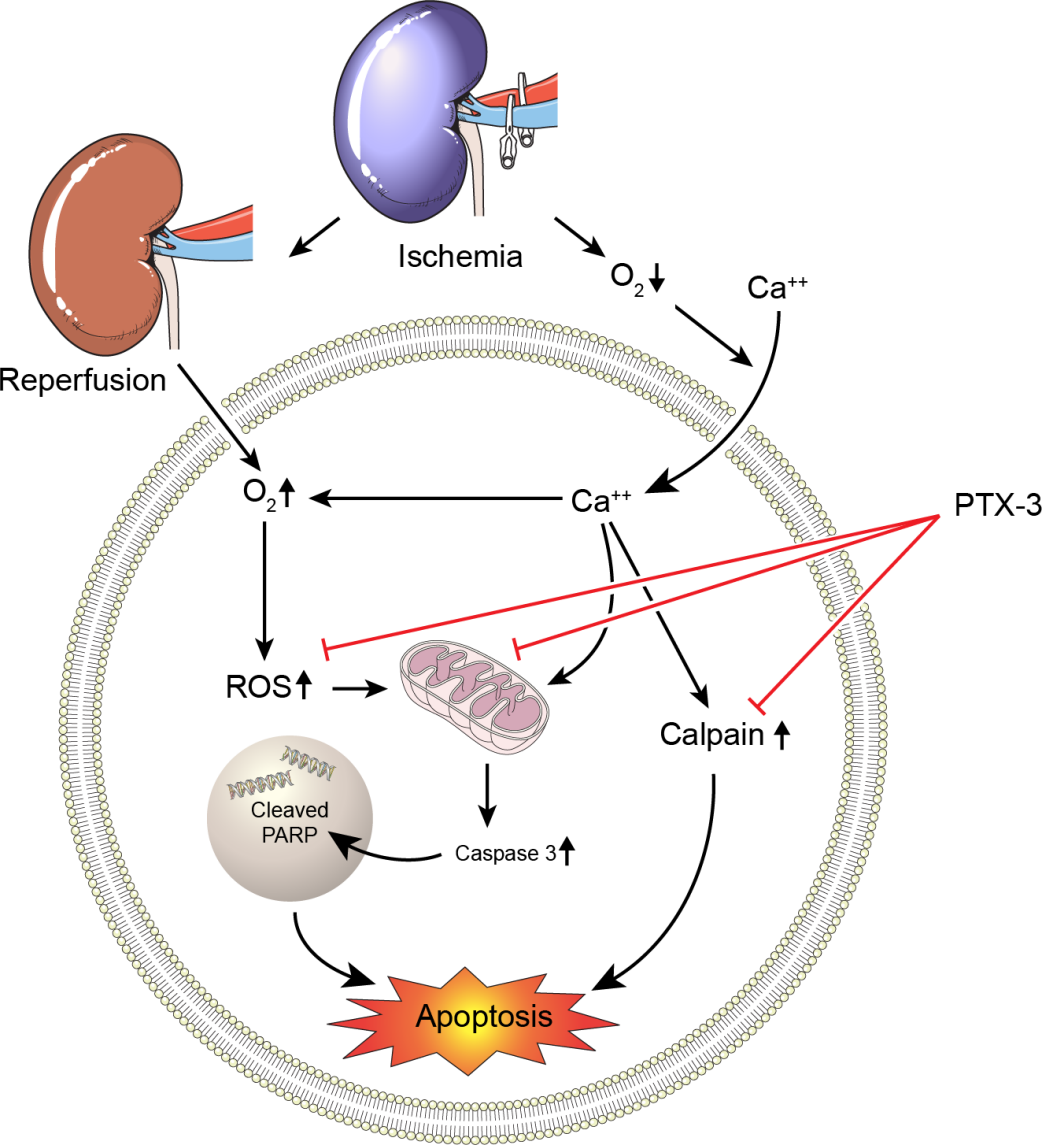
Proposed model of the renoprotective effects of PTX3 on apoptosis-mediated signaling in AKI.

Although the function of PTX3 has been reported to be related to immunity, we identified calpain activity and MMP in kidney cells as targets of PTX3 function (Fig 8). Future studies are needed to determine if PTX3 performs similarly in other models of IRI and whether recombinant PTX3 protects against apoptosis *in vivo*. Such experiments would broaden considerably the translational aspect of recombinant PTX3 and might provide further mechanistic insight into the beneficial properties of PTX3 observed against IRI.

## Conclusions

Our results show that exogenous recombinant PTX3 protects renal cells against IRI and apoptosis. PTX3 reduces the generation of ROS, suppressed calpain and caspase-3 activity, and stabilizes the MMP. Our studies may lead to new therapeutic approaches using a drug that reduces apoptotic signals in renal cells after IRI.

## Supporting information

S1 Table Raw data of Fig 1A. (DOCX)

S2 Table Raw data of Fig 1B. (DOCX)

S3 Table Raw data of Fig 2A. (DOCX)

S4 Table Raw data of Fig 2B. (DOCX)

S5 Table Raw data of Fig 3B. (DOCX)

S6 Table Raw data of Fig 4B. (DOCX)

S7 Table Raw data of Fig 5B. (DOCX)

S8 Table Raw data of Fig 6B. (DOCX)
