# Supplementary material for: Exogenous pentraxin-3 inhibits the reactive oxygen species-mitochondrial and apoptosis pathway in acute kidney injury
Source: PLoS One. 2018 Apr 19;13(4):e0195758. doi: 10.1371/journal.pone.0195758 (PMC5909599; doi:10.1371/journal.pone.0195758)
Supplement: S1 Table — (DOCX) [file pone.0195758.s001.docx]

Table S1. Raw data of figure 1A.

|  | PTX-3 | | | |
| --- | --- | --- | --- | --- |
|  | con | 0.01 | 1 | 5 |
| 1 | 0.8236 | 0.9137 | 0.9964 | 0.9703 |
| 2 | 0.8555 | 0.9548 | 0.9609 | 0.9743 |
| 3 | 0.8326 | 0.9541 | 1.010 | 1.013 |
| Mean | 0.8372 | 0.9408 | 0.9894 | 0.9860 |
| SD | 0.1644 | 0.0235 | 0.0257 | 0.0238 |
