# Supplementary material for: Exogenous pentraxin-3 inhibits the reactive oxygen species-mitochondrial and apoptosis pathway in acute kidney injury
Source: PLoS One. 2018 Apr 19;13(4):e0195758. doi: 10.1371/journal.pone.0195758 (PMC5909599; doi:10.1371/journal.pone.0195758)
Supplement: S2 Table — (DOCX) [file pone.0195758.s002.docx]

Table S2. Raw data of figure 1B.

|  |  | PTX-3 | | | | TGF-b |
| --- | --- | --- | --- | --- | --- | --- |
|  | con | P10 | P100 | P200 | P500 | T50 |
| 1 | 0.9972 | 0.9546 | 0.9315 | 0.9555 | 0.9299 | 1.136 |
| 2 | 0.9767 | 0.9556 | 0.9326 | 0.9564 | 0.9247 | 1.141 |
| 3 | 0.9630 | 0.9638 | 0.9354 | 0.9562 | 0.9322 | 1.130 |
| Mean | 0.9789 | 0.9580 | 0.9331 | 0.9560 | 0.9289 | 1.136 |
| SD | 0.0172 | 0.0050 | 0.0020 | 0.0004 | 0.0038 | 0.0058 |
