# Supplementary material for: Exogenous pentraxin-3 inhibits the reactive oxygen species-mitochondrial and apoptosis pathway in acute kidney injury
Source: PLoS One. 2018 Apr 19;13(4):e0195758. doi: 10.1371/journal.pone.0195758 (PMC5909599; doi:10.1371/journal.pone.0195758)
Supplement: S3 Table — (DOCX) [file pone.0195758.s003.docx]

Table S3. Raw data of figure 2A.

| 24h | con | Only A0.3 | A0.3+P1 | A0.3+P5 | A0.3+P10 |
| --- | --- | --- | --- | --- | --- |
| 1 | 0.9995 | 0.8177 | 0.9295 | 0.9591 | 0.9282 |
| 2 | 0.9457 | 0.8201 | 0.8775 | 0.9147 | 0.8974 |
| 3 | 1.0132 | 0.8492 | 0.8685 | 0.9263 | 0.8808 |
| Mean | 0.9861 | 0.8290 | 0.8918 | 0.9333 | 0.9021 |
| SD | 0.0356 | 0.0175 | 0.0329 | 0.0230 | 0.0240 |

| 48h | con | Only A0.3 | A0.3+P1 | A0.3+P5 | A0.3+P10 |
| --- | --- | --- | --- | --- | --- |
| 1 | 1.2565 | 0.7946 | 0.9695 | 0.9762 | 0.9811 |
| 2 | 1.2751 | 0.7848 | 1.0612 | 1.0113 | 0.9982 |
| 3 | 1.2794 | 0.7594 | 1.0357 | 1.0614 | 1.0125 |
| Mean | 1.2703 | 0.7796 | 1.0221 | 1.0163 | 0.9972 |
| SD | 0.0121 | 0.0181 | 0.0473 | 0.0428 | 0.0157 |
