# Supplementary material for: Exogenous pentraxin-3 inhibits the reactive oxygen species-mitochondrial and apoptosis pathway in acute kidney injury
Source: PLoS One. 2018 Apr 19;13(4):e0195758. doi: 10.1371/journal.pone.0195758 (PMC5909599; doi:10.1371/journal.pone.0195758)
Supplement: S4 Table — (DOCX) [file pone.0195758.s004.docx]

Table S4. Raw data of figure 2B.

|  | con | Only Hypoxia | H+P1 | H+P5 | H+P10 |
| --- | --- | --- | --- | --- | --- |
| 1 | 0.7983 | 0.6760 | 0.6874 | 0.7463 | 0.7380 |
| 2 | 0.8490 | 0.6900 | 0.7729 | 0.7692 | 0.7427 |
| 3 | 0.8029 | 0.6909 | 0.6814 | 0.7438 | 0.7392 |
| Mean | 0.8167 | 0.6856 | 0.7139 | 0.7531 | 0.7399 |
| SD | 0.0280 | 0.0083 | 0.0511 | 0.0139 | 0.0024 |
