# Supplementary material for: Exogenous pentraxin-3 inhibits the reactive oxygen species-mitochondrial and apoptosis pathway in acute kidney injury
Source: PLoS One. 2018 Apr 19;13(4):e0195758. doi: 10.1371/journal.pone.0195758 (PMC5909599; doi:10.1371/journal.pone.0195758)
Supplement: S5 Table — (DOCX) [file pone.0195758.s005.docx]

Table S5. Raw data of figure 3B.

|  | con | Only A0.3 | A0.3+P5 | Only A0.6 | A0.6+P5 |
| --- | --- | --- | --- | --- | --- |
| 1 | 26.352 | 31.605 | 27.572 | 36.738 | 32.957 |
| 2 | 24.628 | 30.489 | 25.943 | 34.874 | 32.057 |
| 3 | 25.617 | 29.348 | 26.193 | 35.757 | 31.073 |
| 4 | 23.778 | 26.107 | 27.198 | 31.581 | 29.919 |
| Mean | 25.093 | 29.387 | 26.726 | 34.737 | 31.501 |
| SD | 1.125 | 2.373 | 0.7822 | 2.237 | 1.305 |
