# Supplementary material for: Exogenous pentraxin-3 inhibits the reactive oxygen species-mitochondrial and apoptosis pathway in acute kidney injury
Source: PLoS One. 2018 Apr 19;13(4):e0195758. doi: 10.1371/journal.pone.0195758 (PMC5909599; doi:10.1371/journal.pone.0195758)
Supplement: S6 Table — (DOCX) [file pone.0195758.s006.docx]

Table S6. Raw data of figure 4B.

|  | con | only A 0.3 | A0.3+P1 | A0.3+P5 |
| --- | --- | --- | --- | --- |
| 1 | 4.47 | 13.82 | 7.22 | 5.58 |
| 2 | 6.56 | 9.75 | 7.34 | 6.23 |
| 3 | 8.69 | 12.85 | 6.69 | 4.66 |
| 4 | 8.44 | 14.32 | 9.14 | 6.93 |
| Mean | 7.040 | 12.685 | 7.597 | 5.850 |
| SD | 1.959 | 2.049 | 1.066 | 0.9660 |
