# Supplementary material for: Exogenous pentraxin-3 inhibits the reactive oxygen species-mitochondrial and apoptosis pathway in acute kidney injury
Source: PLoS One. 2018 Apr 19;13(4):e0195758. doi: 10.1371/journal.pone.0195758 (PMC5909599; doi:10.1371/journal.pone.0195758)
Supplement: S7 Table — (DOCX) [file pone.0195758.s007.docx]

Table S7. Raw data of figure 5B.

|  | con | Only A0.3 | A0.3+P1 | A0.3+P5 | Only A1 |
| --- | --- | --- | --- | --- | --- |
| 1 | 2.6798 | 1.4577 | 0.5439 | 0.3731 | 0.9836 |
| 2 | 3.6476 | 1.9165 | 0.5254 | 0.2741 | 0.7707 |
| 3 | 2.2310 | 1.8284 | 0.8195 | 0.4482 | 0.5966 |
| 4 | 2.7656 | 1.1509 | 0.4161 | 0.3615 | 0.5040 |
| 5 | 2.5355 | 1.3873 | 0.5471 | 0.3943 | 0.6899 |
| 6 |  |  |  |  | 0.7230 |
| 7 |  |  |  |  | 0.5544 |
| 8 |  |  |  |  | 0.6363 |
| Mean | 2.771 | 1.5481 | 0.5704 | 0.3702 | 0.6889 |
| SD | 0.5300 | 0.3186 | 0.1492 | 0.0632 | 0.1610 |
