# Supplementary material for: Exogenous pentraxin-3 inhibits the reactive oxygen species-mitochondrial and apoptosis pathway in acute kidney injury
Source: PLoS One. 2018 Apr 19;13(4):e0195758. doi: 10.1371/journal.pone.0195758 (PMC5909599; doi:10.1371/journal.pone.0195758)
Supplement: S8 Table — (DOCX) [file pone.0195758.s008.docx]

Table S8. Raw data of figure 6B.

|  | con | only A 0.3 | A0.3+P1 | A0.3+P5 |
| --- | --- | --- | --- | --- |
| 1 | 0.0068 | 0.3300 | 0.2312 | 0.1652 |
| 2 | 0.0050 | 0.5446 | 0.2265 | 0.1074 |
| 3 | 0.0074 | 0.3573 | 0.2198 | 0.1345 |
| 4 | 0.0014 | 0.4230 | 0.2020 | 0.0946 |
| 5 | 0.0044 | 0.4464 | 0.0738 | 0.1207 |
| Mean | 0.5068 | 42.02 | 19.07 | 12.45 |
| SD | 0.2359 | 8.407 | 6.624 | 2.719 |
